# Specialist respiratory outreach: a case-finding initiative for identifying undiagnosed COPD in primary care

**DOI:** 10.1038/s41533-021-00219-x

**Published:** 2021-02-11

**Authors:** Emma Ray, David Culliford, Helen Kruk, Kate Gillett, Mal North, Carla M. Astles, Alexander Hicks, Matthew Johnson, Sharon Xiaowen Lin, Rosanna Orlando, Mike Thomas, Rachel E. Jordan, David Price, Mita Konstantin, Tom M. A. Wilkinson

**Affiliations:** 1 NIHR ARC Wessex, Faculty of Health Sciences, University of Southampton, Southampton, UK; 2 University Hospital Southampton NHS Foundation Trust, Southampton, UK; 3 Department of Primary Care and Population Sciences, University of Southampton, Southampton, UK; 4 Institute of Applied Health Research, University of Birmingham, Birmingham, UK; 5 Observational and Pragmatic Research Institute, Singapore, Singapore; 6 Centre of Academic Primary Care, Division of Applied Health Sciences, University of Aberdeen, Aberdeen, UK; 7 Faculty of Medicine, University of Southampton, Southampton, UK; 8 NIHR Biomedical Research Centre, University Hospitals Southampton NHS Foundation Trust, Southampton, UK; 9 Wessex Investigational Sciences Hub, University of Southampton Faculty of Medicine, Southampton General Hospital, Southampton, UK

**Keywords:** Chronic obstructive pulmonary disease, Health care, Medical research, Respiratory signs and symptoms

## Abstract

COPD remains largely undiagnosed or is diagnosed late in the course of disease. We report findings of a specialist outreach programme to identify undiagnosed COPD in primary care. An electronic case-finding algorithm identified 1602 at-risk patients from 12 practices who were invited to attend the clinic. Three hundred and eighty-three (23.9%) responded and 288 were enrolled into the study. Forty-eight (16.6%) had undiagnosed mild and 28 (9.7%) had moderate airway obstruction, meeting spirometric diagnostic criteria for COPD. However, at 12 months only 8 suspected COPD patients (10.6%) had received a diagnostic label in their primary care record. This constituted 0.38% of the total patient population, as compared with 0.31% of control practices, *p* = 0.306. However, if all patients with airway obstruction received a coding of COPD, then the diagnosis rate in the intervention group would have risen by 0.84%. Despite the low take-up and diagnostic yield, this programme suggests that integrated case-finding strategies could improve COPD recognition.

## Introduction

Chronic obstructive pulmonary disease (COPD) is a common, serious and disabling lung condition, usually caused by regular exposure to noxious inhaled agents, particularly tobacco smoke^1–3^. It is a preventable and treatable disease, although it is usually progressive once established^2,4,5^. It is characterised by persistent airflow obstruction and persistent respiratory symptoms, including dyspnoea, cough and sputum production, with episodic acute exacerbations^1,6,7^. Guidelines recommend that the diagnosis should be considered in any patient with suggestive symptoms and a history of exposure to relevant risk factors, such as cigarette smoke and indoor biomass cooking fumes. Persistent obstruction post bronchodilator needs to be demonstrated to confirm the diagnosis^1,2^. There is, however, consistent and ongoing evidence of major under-diagnosis of COPD globally including in the UK^8–10^. Based on epidemiological surveys, it is estimated that the true global prevalence of COPD is 11.7% (95% confidence interval (CI) 8.4–15.0%), yet the proportion of the population told that they have COPD is <6% in most national data sets, and often as low as 2–3%, reflecting widespread under-recognition and under-diagnosis. The reasons for under-diagnosis are varied: some patients may not seek medical care due to mild symptoms, some may tolerate symptoms rather than seeking help or may choose to self-manage, and some may have difficulty accessing medical care^11–14^. However, many patients will consult with their primary care clinicians for persistent respiratory symptoms, or for acute episodes, representing unrecognised exacerbations, and may receive treatment (e.g. antibiotics, bronchodilators, corticosteroids, antitussives) without a diagnosis being made or diagnostic investigations for the underlying problem undertaken^15^.

Although COPD is not curable, there are a number of cogent reasons for encouraging an early diagnosis. First, as a preventable progressive condition, recognition of early disease allows targeting of preventative measures to reduce disease progression, notably interventions to reduce exposures, such as smoking cessation and reduction of exposure to airborne pollutants, including indoor biomass fuel fumes^2,16^. There is some evidence to suggest that demonstration of lung damage by spirometry can support the motivation and success of smoking cessation^17^. Other relevant lifestyle and prevention measures for patients with early COPD include diet and exercise advice and appropriate vaccination and referral for pulmonary rehabilitation (PR) offered in a variety of models where appropriate^1,18–23^. Additionally, although pharmacotherapy is not curative, effective medication can reduce symptoms, exacerbations, lung function decline and mortality as well as improving and quality of life^1,2,24^.

Although guidelines agree on the desirability of early diagnosis, the best way to achieve this is unclear. A US systematic review concluded that population-level screening with spirometry in asymptomatic adults was not a viable strategy logistically, or economically, but did encourage clinicians to ‘pursue active case-finding for COPD in patients with risk factors or respiratory symptoms^25^. All active case-finding initiatives rely on identification of those at risk of COPD for screening, and a number of approaches have been investigated, including targeting smokers with screening questionnaires and then inviting those screening positive to attend for diagnostic spirometry^26–32^. Several COPD risk-prediction models have been proposed^33,34^, but variations in methods and a lack of prospective randomised controlled trials to confirm the viability, clinical and cost effectiveness of these approaches has hindered adoption into routine clinical practice. In the UK, a COPD risk score for identifying currently undiagnosed patients based on information present in the routine primary care clinical record (the TargetCOPD score) has recently been developed and successfully implemented^35^. This algorithm uses individual patient information from the medical record on age, smoking status, dyspnoea consultations, prescriptions for salbutamol and prescriptions for antibiotics to generate an individual COPD risk score and has the potential to be electronically automated and applied to primary care electronic records.

In the current pressurised clinical climate, many primary care centres are stretched in coping with rising clinical workload and may be reluctant to take on additional screening activities; an alternative approach is using ‘interface’ teams, such as outreach specialist groups, to support general practice (GP) and provide integrated care between hospital and community-based sectors. Such initiatives do, however, need to be prospectively evaluated in pilots for acceptability, clinical and cost effectiveness prior to wider implementation.

We report the prospective analysis of a pilot clinical service set in routine UK GP, using the TargetCOPD score applied electronically to the primary care routine electronic medical record, to identify currently undiagnosed patients at risk of COPD and invite them for diagnostic assessment at their GP practice by a specialist outreach nursing team with feedback to practices. An assessment of the new COPD diagnosis rate in participating centres in the year following the intervention was made, with comparison to the diagnosis rate in similar patients receiving usual care in matched control practices over the same time interval (Supplementary Methods).

## Results

### Main results

Twelve practices were included in the study, with list sizes of 8196–15,422, combined total patient population 147,673, with a mean (range) prevalence of diagnosed COPD prior to the study of 1–3%.

**Fig. 1:**
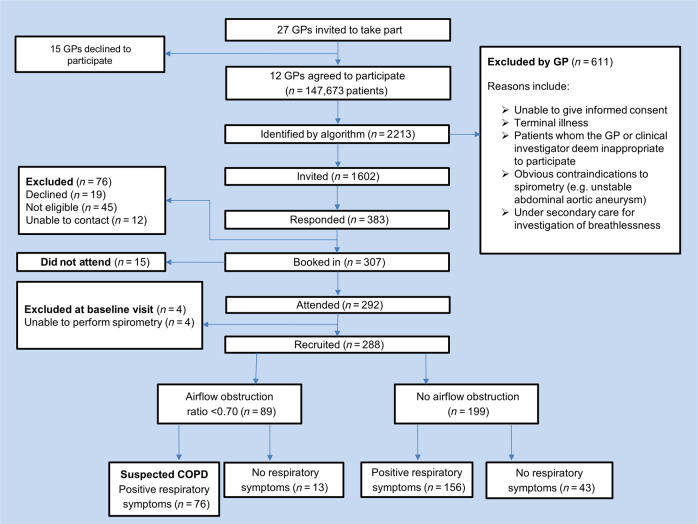
Patient flow through the study. Indicated is the recruitment of general practices and patients. It also shows the number of GP practices that agreed to participate and the number of patients identified, invited, responded, booked and recruited. Numbers of patients determined to have airflow obstruction by spirometry and respiratory symptoms are also displayed.

The electronic score identified 2213 at-risk patients (Fig. 1). The lead GPs assessed the list and excluded 611 patients considered inappropriate to invite for assessment. One thousand six hundred and two patients were sent postal invitations to attend for assessment, and 383 (23.9%) patients responded. Seventy-six patients who responded were not included in the study because they declined to participate, were uncontactable or were found to be not eligible. Four further patients were excluded because they were unable to perform spirometry and 15 patients did not attend their booked appointment. In total, 288 (male 51%, mean age 63, standard deviation (SD) = 6.71 years) attended the case-finding clinic and provided informed consent. Of these, 76 (26.4%) met the UK spirometric diagnostic criteria for COPD (post-bronchodilator ratio <0.70 and concurrent respiratory symptoms) of whom 48 (63.1%) had mild airflow obstruction (GOLD 1: percentage of forced expiratory volume in 1 s (FEV_1_%) predicted ≥80%), 28 (36.8%) moderate (GOLD 2: FEV_1_% predicted ≥50–<80% airflow obstruction) and none had severe/very severe airflow obstruction. In a sensitivity analysis using age-sex specific ‘lower limit of normal’ (LLN) criteria (Global Lung Initiative equations^36^), fewer patients met diagnostic criteria for COPD (39, 13.5%).

In comparison with those without airflow obstruction, the patients with suspected COPD reported more chronic cough (52, 68.4% versus 110, 51.9%, Chi-squared test, *p* = 0.013), dyspnoea (42, 55.3% versus 98, 46.2%, Chi-squared test, *p* = 0.176), wheeze (43, 56.6% versus 75, 35.4%, Chi-squared test, *p* = 0.001) and chronic sputum (29, 38.2 versus 60, 28.3%, Chi-squared test, *p* = 0.111). Fifty patients (66.7%) with suspected COPD and 108 (50.9%, Chi-squared test, *p* = 0.019) of patients with no suspected COPD reported significant dyspnoea related to physical activity (Medical Research Council dyspnoea (MRC) scale ≥2). Among breathless patients (MRC ≥ 2), there was no difference in mean body mass index (BMI) between those with suspected COPD and those without (mean, SD: 29.5 (6.2) versus 30.9 (5.8), 95% CI for difference: [−0.7, 3.4], *t* test, *p* = 0.209), with only 3 out of 50 and 5 out of 108, respectively, being morbidly obese (BMI ≥ 40).

A previous diagnosis of asthma was more common in patients with suspected COPD (34, 44.7% versus 51, 24.1%, Chi-squared test, *p* = 0.001). Current smoking was more common in patients with suspected COPD than without (23, 30.3% versus 43, 20.3%, Chi-squared test, *p* = 0.079) as was a higher mean pack-year history (29.3, interquartile range (IQR) [15, 45] versus 17.4, IQR [7, 33.5], Mann–Whitney *U* test, *p* < 0.001). Mean FEV_1_ was also lower in those with suspected COPD (2.38 L, SD 0.66 versus 2.75 L, SD 0.68, *t* test, *p* < 0.001).

### New COPD diagnoses in the subsequent 12 months

In the year following the intervention in the active practices, 57 patients received a new diagnosis of COPD in their medical record from a base population of 14,818 ever smokers in the 40–79-year age group, equating to 0.38%. In the control practices, there were 46 new diagnosis in 14,638 (0.31%), (Chi-squared test, *p* = 0.306). This equates to a COPD new diagnosis rate per 1000 patients in the overall practice list of 3.84 in the active and 3.14 in the control practices. Further analysis of the newly diagnosed cases in the active practices revealed that only 8 of the 76 patients with obstructive spirometry on assessment and suspected COPD subsequently received a new diagnosis of COPD recorded in the electronic medical record with the other 49 new diagnoses made in patients who did not attend the diagnostic assessments.

### Other effects of the programme

In addition to new diagnosis, we assessed other clinical consequences of the assessment in the active practices. Recommendations for further action by the GPs were made in 112 (38.9%) patients, 61 (80.3%) of patients with suspected COPD and 51 (24.0%) of patients with no COPD. This included smoking cessation, weight loss clinics and referral to respiratory specialist medical teams. For those with suspected COPD (*N* = 76, 26.4%), the study team recommended further clinical investigations that included: chest X-rays (56, 73.7%) and electrocardiogram (11, 14.5%), sputum cultures (4, 5.3%) and bloods tests (34, 44.7%) and specialist referral (*n* = 22, 28.9%). Treatment recommendations were made for 119 (41.3%) patients, the majority being inhaled medications with adjuncts (spacer devices), plus intranasal steroids in 7 cases while 2 patients were recommended a proton-pump inhibitor.

All active smokers (*n* = 66) received brief smoking cessation advice with an offer of specialist smoking service referral, which was accepted by 6 (26.1%) patients with suspected COPD versus 18 (41.9%) of patients with no COPD. Referrals to a weight loss clinic were accepted by more patients with no COPD than those with suspected COPD (12, 5.6% versus 2, 2.6%) despite BMI being similar between groups (Table 1). PR referral was agreed with 12 (15.8%) patients with suspected COPD and with 3 (1.4%) patients with no COPD for reasons including bronchiectasis.

**Table 1:** Patient characteristics.

Variable	All observations		Suspected COPD		COPD not suspected		Missing values	*p* value^c^
Participants, *n* (%)	288	(100.0)	76	(26.4)	212	(73.6)		
Age in years, mean (SD)	63.1	(6.7)	63.6	(6.0)	62.9	(7.0)		0.421
Male, *n* (%)	146	(50.7)	43	(56.6)	103	(48.6)		0.232
Ethnicity, *n* (%)								0.394
Caucasian	283	(98.3)	74	(97.4)	209	(98.6)		
Asian	2	(0.7)	1	(1.3)	1	(0.5)		
Afro-Caribbean	1	(0.3)	0	(0.0)	1	(0.5)		
Other	2	(0.7)	1	(1.3)	1	(0.5)		
BMI kg/m^2^, mean (SD)	29.2	(5.3)	28.5	(5.6)	29.5	(5.2)		0.188
Practice IMD decile (median, IQR)	9	(8,10)	9	(8,10)	9	(8,10)		n/a
Smoking status (*n*, %)								0.076
Current smoker	66	(22.9)	23	(30.3)	43	(20.3)		
Ex-smoker	222	(77.1)	53	(69.7)	169	(79.7)		
Smoking pack years (median, IQR)	20	(8,36)	29.3	(15,45)	17.4	(7,33.5)		<0.001
Symptoms, *n* (%)								
Cough	162	(56.3)	52	(68.4)	110	(51.9)		0.013
Dyspnoea	140	(48.6)	42	(55.3)	98	(46.2)		0.176
Wheeze	118	(41.0)	43	(56.6)	75	(35.4)		0.001
Sputum	89	(30.9)	29	(38.2)	60	(28.3)		0.111
MRC breathlessness score, *n* (%)	287		75		212		1	0.060
1	129	(44.9)	25	(33.3)	104	(49.1)		
2	119	(41.5)	34	(45.3)	85	(40.1)		
3	27	(9.4)	12	(17.4)	15	(7.1)		
4	11	(3.8)	4	(5.3)	7	(3.3)		
5	1	(0.3)	0	(0.0)	1	(0.4)		
FENO (median, IQR)	21	(13,33)	22	(12,36)	21	(14,33)	64	0.568
FEV_1_ litres, mean (SD)	2.65	(0.70)	2.38	(0.66)	2.75	(0.68)	3	<0.001
FEV_1_ % predicted (median, IQR)	99	(87,109)	87.5	(74,98)	104	(92,113)	3	<0.001
FEV_1_/FVC ratio (median, IQR)	74	(69,79)	65	(60,68)	77	(73,80.5)	3	<0.001
FEV_1_/VC ratio (median, IQR)	76	(70,81)	66	(59.5,69)	79	(75,83)	19	<0.001
FEF 25–75% predicted (median, IQR)	62	(45.5,82)	38	(27.3,46)	71	(57,90)		<0.001
Co-morbidities, *n* (%)								
Respiratory tract infection in the past 12 months	67	(23.3)	18	(23.7)	49	(23.1)		0.919
Asthma	85	(29.5)	34	(44.7)	51	(24.1)		0.001
Depression or anxiety	70	(24.3)	17	(22.4)	53	(25.0)		0.646
Gastro-oesophageal reflux disease (GORD)	39	(13.5)	8	(10.5)	31	(14.6)		0.371
Endocrine disease^a^	60	(20.8)	20	(26.3)	40	(18.9)		0.170
Cardiac disease^b^	49	(17.0)	12	(15.8)	37	(17.5)		0.741
Chronic rhino-sinusitis	16	(5.6)	2	(2.6)	14	(6.6)		0.195
Allergic rhinitis	16	(5.6)	7	(9.2)	9	(4.2)		0.105
Pneumonia	8	(2.8)	3	(3.9)	5	(2.4)		0.470

Data are presented as *n* (%), mean (standard deviation) or median (interquartile range), unless otherwise stated.

*IMD* index of multiple deprivation (a weighted standardised measure of socioeconomic status), *BMI* body mass index, *FEV*_*1*_ forced expiratory volume in 1 s, *FVC* forced vital capacity.

^a^Endocrine disease is defined as either of diabetes mellitus or hypothyroidism.

^b^Cardiac disease is defined as any of ischaemic heart disease, hypocholesterolaemia, myocardial infarction or atrial fibrillation.

^c^All *p* values computed using tests appropriate for the type and distribution of each variable: *t* test for age, BMI, FEV_1_ (litres); Mann–Whitney *U* test for smoking pack-years, FEV_1_ %predicted, FEV_1_/FVC ratio, FEV_1_/VC ratio, FEF 25–75 %predicted; Chi-squared test for sex, ethnicity, smoking status, MRC breathlessness score, all symptoms and all comorbidities.

### Cost analysis

The mean total costs (primary and secondary care attendances) for respiratory-related issues per patient for the 12 months prior to the baseline visit and at the 12 months follow-up point was collected. In total, 33 patients were lost to follow-up at the 12-month follow-up point. At the baseline visit, health care costs for the previous 12 months were lower for those with suspected COPD (£43.69) versus those with no COPD (£50.50) (see Table 2). Costs in the group with no COPD was driven by 6 outliers who had more frequent contact with the 111 NHS health-support helpline and Emergency Department episodes. When we excluded these outliers, the costs were similar between the groups: no COPD (£45.31) versus suspected COPD (£43.69). In the 12 months following baseline, mean costs were higher in the suspected COPD group (£60.42 versus £46.15, Mann–Whitney *U*, *p* = 0.001), reflecting the cost of additional tests and referrals.

**Table 2:** Summary of patient costs.

	No COPD	Suspected COPD
Baseline number of patients	212	76
Pre-baseline 12-month cost (mean)	£50.50	£43.69
Follow-up number of patients	190	65
Follow-up total cost (average)	£55.21	£60.42

The costs of providing the intervention, excluding OPC costs, were estimated as £57.19 per patient assessed, excluding premises overhead costs and consumables. This is based on the costs of providing the appropriately skilled nurse (UK salary Band 6) conducting the clinic appointment, lasting 1 h 20 min per patient including face-to-face and administrative time. With 288 patients participating in the study, the total staff cost for the intervention was £16,470. This equates to cost per new COPD diagnosis in the practice of £288.90 and costs per patient with confirmed post-bronchodilator airflow obstruction at assessment of £216.70.

### Patient feedback

Patient feedback about the case-finding clinic was sought via a self-reported questionnaire. In total, 285 were included in this analysis and 3 were lost to follow-up. Many (161, 57.5%) reported concerns on receiving the invitation, although most (147, 53.3%) were not surprised to receive it. Despite all patients (100%, *n* = 285) reporting feeling concerned about attending the clinic, 99% (283) reported that attending the clinic was a positive experience and 80% (223) reported that it had made them think about their health and 94.3% (265) reported feeling supported by their GP practice. Further in-depth analysis on patient’s feelings and experiences of attending the clinic has been captured in the qualitative sub-study^37^.

## Discussion

Despite calls for identifying undiagnosed COPD patients earlier in order to instigate preventative and therapeutic interventions to improve health and retard disease progression, the optimal way of achieving this aim remains unclear^9^. This is particularly true when health resources are limited and primary care teams under pressure^38^. A viable focus for COPD case finding is to concentrate on the demographic group with relevant exposures who are already known to the medical system and who are receiving treatments for acute and on-going respiratory episodes that plausibly could relate to undiagnosed COPD^2^. In this initiative, we aimed to prospectively evaluate the effectiveness and acceptability of a specialist outreach team identifying and assessing patients in the community in collaboration with their usual primary care health team. The aim of the initiative was not to replace but to augment the usual primary care service and to provide a convenient and seamless clinical service to at-risk patients.

In our approach, we electronically applied a previously internally validated risk score to routine computerised health records in primary care to identify possible undiagnosed COPD cases^39^. A total of 1602 patients were identified and felt to be appropriate by the GP and received an invitation letter to attend the clinic, of whom 24% of patients invited responded positively and 18% attended and were assessed. The response rate reported in our study is similar to that of other well-reported case-finding trials for COPD^8,35,38^. While it is difficult to make comparisons to other conditions because of the unique study design and disease focus, the response rate in case finding for COPD studies is markedly lower when compared to diabetes case-finding research studies for example. Indeed, Snijder et al. and Greaves et al. reported 55 and 60.6% response rates consecutively to invitation for diabetes screening using case-finding methods^40,41^.

Possible reasons for the low response rate in our research may be due to denial of symptoms particularly if the symptoms are mild and not limiting daily activities. People may also avoid addressing their lung health due to the potential stigma of having a chronic lung disease caused by their tobacco dependency^42,43^. Potential cost implications such as an increase in life and travel insurance, as well as the cost of prescriptions may also be a factor to decline participation^44^. Another barrier might be that smokers who may not be susceptible to quitting may be concerned that they will receive judgemental and negative smoking cessation advice from the health care professional screening them^45^. Patients who responded and were enrolled into the study were generally older and possibly found it more convenient to attend a potentially lengthy appointment, and younger working patients may have had barriers to attendance and so been under-represented. Future clinics could potentially offer appointments in the evenings and at the weekend. Further research should also provide invitees the opportunity to explain why they do not want to attend so that we can better understand the barriers to uptake.

Of those attending for assessment, 26% had post-bronchodilator obstruction on quality-assured spirometry and respiratory symptoms, consistent with COPD. However, only 57 patients in the practices received a diagnosis of COPD in the medical records over the next 12 months, and only 8 of these new diagnoses came in the group undergoing assessment. Consequently, only 1 in 10 of the patients with suspected COPD on assessment received a subsequent COPD diagnosis from their primary care team. The reasons why so few of those identified by the assessment received a diagnosis are not clear and should be a focus in future research, but previous studies have also noted this issue.

It is noteworthy that the majority of cases identified as suspected COPD had mild airflow obstruction, with a lower proportion meeting ‘LLN’ criteria for COPD, implying that an older and relatively mild, early COPD population were being identified. It is plausible that the primary care teams were reluctant to apply the diagnostic label of COPD to these milder cases because of the perceived extra workload and perception that the current COPD caseload is already not being managed well^46^. We quantified the new COPD diagnosis rate in control practices, which was slightly lower than that in our active practices (0.31 versus 0.38%), implying that there is a small but detectable incremental benefit on diagnostic rates of the outreach programme. If all 76 of those who were suspected of having COPD on assessment had received a coding of COPD, then the diagnosis rate in the intervention group would have risen from 0.38% to 0.84%. However, as practices were not randomised to the intervention or control arm, we also cannot be sure that there were no confounding factors at either practice or individual level. The assessment-to-diagnosis yield of 27% we achieved is that predicted from the diagnostic score used, with the number of diagnostic assessments needed per case detected being estimated as 4 (3–7), at the risk threshold of 22.5% used to select patients^33^.

In addition, we found that there were a number of potentially beneficial health interventions made for participants in the programme in addition to making a new COPD diagnosis. These included health promotion interventions such as smoking cessation, dietary interventions and PR, as well as recommendations for further assessment in case of diagnostic uncertainty. These potential benefits occurred in the group with no COPD as well as in those with suspected COPD, who were the focus of the project.

In terms of acceptability to patients, the programme was generally well received; although many patients reported an initial anxiety on receiving the invite, most who attended found the process valuable and beneficial. This reflects the findings of a qualitative sub-study that was conducted to understand the barriers to acceptance of diagnostic case finding in at-risk patients in primary care and is reported elsewhere^37^. Although not formally assessed, the GPs, nurses and administrative staff in the participating practices also found the programme valuable and appreciated the support provided by the visiting team. The intervention had associated staffing costs, which we calculated as being approximately £220 per case of suspected COPD identified, which needs to be balanced against the potential cost savings of better management of the newly diagnosed COPD cases and indeed the health benefits of other patients undergoing assessment. These were not possible to estimate in this study.

There are, however, a number of factors that limit the potential of using this model in wider settings. First, as only a quarter of those identified by the score and were invited to attend took up the opportunity, this implies that, even when provided in a local setting, such a service may not be attractive to the majority of at-risk patients. An alternative approach might be that practices independently run the case-finding algorithm on their clinical database in order to identify patients at risk of having COPD. They could then either invite patients to attend for diagnostic spirometry or add a flag on the patients’ records to allow for future opportunistic screening for COPD, so that when they subsequently attend for medical care (possible for respiratory problems), the attending clinicians are made aware that this patient is at risk and may need further investigations beyond the treatment of the presenting acute episode.

Second, although the information on the assessment was fed back to the practice staff, only 8 of the 76 patients (10.6%) with post-bronchodilator obstruction at assessment subsequently received a primary care diagnosis of COPD, so further work is needed to understand the reluctance to formally apply a diagnostic label COPD and the effects of the assessment on the appropriateness of their subsequent management. Despite the study team communicating individual findings with the GP practices, it is possible that intense workload pressures in primary care may have impeded documentation of a formal diagnosis^46,47^. In addition, while the GP practice record is a rich source of clinical data, it is primarily for care purposes, and thus when used in research settings can result in inaccuracies and complications with analysis^47^. Clinicians in our project may plausibly have been reluctant to assign the diagnostic term COPD because of the potential impact to the individual and to the practice workload^48^. Additionally, the onus was placed on the patient to make a follow-up appointment with the GP to discuss the findings, therefore if they did not attend the opportunity to confirm the diagnosis may have been missed.

In conclusion, we report the results of a specialist outreach programme to identify undiagnosed COPD in primary care by generating an ‘at-risk’ list by the electronic application of a validated screening score to the computerised routine patient records and inviting those identified to an assessment by a visiting specialist nursing team in their usual GP surgeries. We found a low response rate, with approximately a quarter of identified at-risk patients attending the assessment, but those who attended found it valuable. For most patients, some health actions were taken, particularly health promotion activities. In total, 76 of those assessed had post-bronchodilator airflow obstruction consistent with COPD, although most had mild obstruction, and a lower proportion met ‘LLN’ criteria for COPD. Only 10% of those with airflow obstruction subsequently received a primary are diagnostic label, for reasons that are not clear. The overall new COPD diagnostic rate in the participating practices was slightly (but not statistically significant given the sample size) higher than that in control practices, implying that implementation of the programme could result in a small benefit to COPD new diagnosis rate. Although the results do not justify wider implementation of this model of case finding in its current format, further investigation of the use of ‘interface’ specialist outreach services and of electronic risk-profiling using primary care medical records is justified; however, the longer-term benefits to these programmes need to be established at the outset.

## Methods

### Study design

The specialist team comprised of respiratory nurse specialists based at Southampton General Hospital, UK, with clinical support from hospital chest physicians and a General Practitioner with a specialist interest in respiratory medicine. In total, 12 local GP surgeries agreed to participate in the programme from rural and urban settings and a broad range of deprivation profiles. The practices agreed for the team to apply the algorithm to the electronic patient medical records, to send mailed invitations for screening at the surgery for those identified as being at risk, to provide use of their premises for the diagnostic sessions and to receive information and recommendations from the outreach team. The outreach team did not make a definitive diagnosis or initiate treatment but provided the usual clinical team with the results of their assessments and any recommendations for treatment, further investigation or referral.

The electronic record search was made by Optimum Patient Care (OPC), a non-profit social enterprise working with primary care to improve the management of patients with chronic diseases through quality improvement programmes and ethically approved research (https://optimumpatientcare.org). Lists of Read Codes related to the risk score were collated by the study team with support from primary care physicians and provided to OPC in order to complete the search. A COPD risk threshold of 22.5% as estimated by the algorithm was implemented based on the original modelling by the Target COPD team and following piloting of a 25% cut-off, which identified higher numbers of patients but less cases of COPD^39^. We therefore determined that the 22.5% cut-off would provide the best positive predictive value to more accurately identify patients with COPD to balance the risk of ‘missing’ people who did have COPD, versus minimising the proportion of ‘false positives’ and to make the intervention efficient and acceptable, minimising unproductive staff workload, as well as the potential inconvenience and anxiety caused to patients.

The identified population was then sent a postal invitation to attend for assessment with an explanation of the study (Supplementary Fig. 1). All eligible participants who responded to invitation by post attended a 60-min case-finding clinic at their GP practice, where the outreach team nurses obtained written informed consent for anonymised data to be used in the prospective analysis, obtained a clinical history, administered questionnaires and performed quality-assured spirometry. Study eligibility criteria included being registered with the participating practices from 1 January 2015 or before, age ≥40 and ≤79 years, no previous diagnosis of COPD, smoker or ex-smoker and able to complete spirometry testing. The assessment consisted of demography, height, weight, blood pressure, medical and smoking history, spirometry with reversibility to 400 µcg of salbutamol via a metered dose inhaler and spacer device (Microlab^®^ spirometer, ERS standards), fractional exhaled nitric oxide (NIOX Vero^®^) and oxygen saturation (Nonin Onyx Vantage^®^). To reduce the potential impact that smoking tobacco has on the lung function tests, we asked patients to refrain from smoking prior to the appointment and accepted patients’ self-reported non-smoking status. Questionnaires completed were: MRC scale^49^ and EuroQOL (EQ-5D) questionnaire^50^. Additionally, a patient experience feedback questionnaire was designed to capture participant views on being invited and attending the clinic. At the end of the appointment, the findings were discussed with the patient and brief health advice on smoking, diet and exercise provided where appropriate. When COPD was suggested, or diagnostic uncertainty existed, patients were asked to arrange a follow-up appointment with their GP. The team aimed to hold meetings with the GP and practice nurse to discuss findings, and when not possible, a full written summary was provided, including recommendations for treatment, further investigations or referral. Recruitment occurred between January 2017 and January 2018. Those attending were followed up with postal questionnaires at 3 and 12 months.

Ethics approval was provided by Southampton B Ethics Committee (16/SC/0629), and the trial is registered on ClinicalTrials.gov (ID: NCT03355677).

### Data analysis

To estimate the incremental diagnostic yield produced by the intervention over usual care, we quantified the new COPD diagnosis rate in matched control practices. Control practices were drawn from the OPC practice portfolio, matched to active sites using practice list size, baseline COPD and asthma diagnostic register rates, gender, age and index of multiple deprivation profiles (Supplementary Table 1). Other than anonymised data extraction, no additional clinical care or feedback to the clinical team occurred in the control practices. Using pseudo-anonymised data, the algorithm was applied electronically in the control practices to identify undiagnosed patients with similar risk profiles as in the active practices, and the COPD diagnosis rate in these patients over the next 12 months was ascertained from the medical record.

### Statistical analysis

Summary statistics are reported for demographic data. Comparisons between the active and control groups were made using Mann–Whitney *U* test or *t* test as appropriate for continuous variables and the Chi-squared test for categorical variables, using a two-sided 5% significance level. Statistical analyses were performed using R (R Core Team, R Foundation for Statistical Computing, Vienna, Austria) and SPSS version 24 (SPSS software, IBM Corp., Armonk, NY). A comparison of health care costs for respiratory issues in the 12 months preceding and in the 12 months following the intervention was made in the study patients undergoing assessment, including primary care costs (GP and nurse appointments, investigations) and secondary care costs (outpatient, A&E, inpatient), using patient recall at the baseline visit and at the 12 months. Unit costs were referenced from the ‘National Schedule of Reference Costs - Year 2015-16 - NHS trusts and NHS foundation trusts’ and adjusted for the relevant time.

### Reporting summary

Further information on research design is available in the Nature Research Reporting Summary linked to this article.

## Supplementary information

Supplementary Information

Reporting Summary

## Data Availability

All data generated or analysed during this study are included in this published article [and its supplementary information files]. Further clarification of results are available upon request to the authors.
