# Supplementary material for: Specialist respiratory outreach: a case-finding initiative for identifying undiagnosed COPD in primary care
Source: NPJ Prim Care Respir Med. 2021 Feb 11;31:7. doi: 10.1038/s41533-021-00219-x (PMC7878732; doi:10.1038/s41533-021-00219-x)
Supplement: Supplementary file 1 — Supplementary Information [file 41533_2021_219_MOESM1_ESM.pdf]

Supplementary Figure 1

Southern Health NHS Foundation Trust
CLAHRC Wessex
University Hospital Southampton NHS Foundation Trust
NHS West Hampshire Clinical Commissioning Group
University of Southampton

The ASSIST Study

Participant Information Sheet

The ASSIST Study

A clinical interventional study into Airways diSease caSefInding and at riSk case managemenT

Case Finding

What is the purpose of the study?
Current research shows that many people in the UK today could be suffering from a lung disease yet not know about it.

The aim of the study is to find patients on a GP practice register that may be at higher risk of developing a lung condition called Chronic Obstructive Pulmonary Disease (COPD). COPD is an ‘umbrella’ term used to describe emphysema and chronic bronchitis which affects 1 person in 20 in the UK and is often associated with a history of smoking or exposure to work related dusts or gases. We would also like to see if patients benefit in the longer term by having a lung health check at their own GP practice with lung health specialists.

Why have I been invited?
You have been invited to take part in this study because signs and symptoms in your medical history may suggest you could have a lung health condition.

What do I have to do?
Once you have read and understood this information sheet, if you agree to take part in the study you will be offered an appointment at your GP practice with a member of the clinical research lung specialist team and the Practice Nurse may also attend. The appointment will last approximately 90 minutes.

Visit 1 – Lung Health Check Visit
Things to bring with you

- This information sheet
- A list of your current medications
- Spectacles if you need them for reading.

Things to avoid before you come to the clinic

- Please avoid having anything to eat for 2 hours prior to the visit
- You may continue to drink, but please avoid caffeinated and hot drinks on the morning of the visit (eg: tea, coffee, coca-cola and energy drinks)
- Please avoid smoking for at least 24 hour prior to the visit (if applicable)
- Please avoid strenuous exercise at least 1 hour prior to the visit

What will happen at the visit?

- At your first visit you will need to sign a consent form. This allows the research team to gather information about you to use in the study. It also confirms that you understand what the study is about and that all your questions have been answered to your satisfaction. We have attached a copy of the consent form so that you know what you will be asked.
- You will then be asked about yourself and your lifestyle, your medical problems you may already have and about any medication you may currently be taking.
- We will take some measurements from you. This will include your oxygen levels, blood pressure, heart rate, breathing rate, height and weight.
- The research team will then carry out two simple breathing tests. The first test will measure the amount of a certain gas that is found in the lungs. For this you will need to blow steadily into a machine for about 10 seconds. The second test is called spirometry which will measure how fast you can empty your lungs and how big your lungs are. For this test you will need to blow into a machine as hard and as quickly as you can.
- After the breathing tests you will be asked to use a blue 'reliever' inhaler which contains a medication called Salbutamol. This is commonly used by people with lung disease to open up their airways in their lungs to make breathing feel easier. To do this we will attach a Salbutamol inhaler to a spacer device. After using the inhaler you will be asked to wait about 15 minutes before repeating the spirometry breathing test. This will allow us to measure how your lungs are after being given the inhaler. After the test you will be asked if you would like information about the results. If you would then we will talk you through the results, however, if the results do show any changes we cannot give you a full diagnosis until all the information is reviewed together by your GP who will then contact you.
- While you are waiting to repeat the test you will be asked to complete some questionnaires about your current health, how active you are and any symptoms you may be experiencing.

Visit Two – Questionnaires – 3 months

Approximately 3 months after your first visit you will be sent a questionnaire to complete at home. This should take about 5 minutes to complete. A pre-paid envelope will provided, so that you can return it to the study team once they have been completed. If you do not return the questionnaire to the study team within three weeks, the study team will contact you to see if you still wish to participate in the study and to offer any assistance with completing the questionnaires.

Visit Three – Questionnaires – 12 months

Approximately 12 months after your first visit you will again be sent some questionnaires to complete at home. These should take about 15 minutes to complete. A pre-paid envelope will be provided, so that you can return them to the study team once they have been completed. If you do not return the questionnaires to the study team within three weeks, the study team will contact you to see if you still wish to participate in the study and to offer any

assistance with completing the questionnaires. Once the study team receives the questionnaires they will then notify your GP that you have completed the study.

Telephone interview
A small number of patients will be interviewed to find out more about their experience around taking part in the study. Interviews will be carried out by a student researcher from the University of Southampton who is working with the CLAHRC team. If you do not want to be interviewed, you can say no. If you agree to be interviewed, you will be interviewed over the telephone at a time convenient to you. We will audio record the interview and it is likely to take 30-60 minutes. Even if you agree to do the interview now you can still decide not to take part when the time comes. The interviews once recorded will then be transcribed into a written format by the student researchers.

What are the advantages and disadvantages of taking part?
Potential advantages: The study has the potential to identify people with a new diagnosis of a respiratory condition, such as COPD. If you do have a lung condition then you may be experiencing symptoms and this may be affecting your life. Early identification of lung conditions means that we can offer treatment, support and education to help keep you well and reduce or minimise your symptoms. Research shows that the earlier that we can identify a lung condition the better the outcome may be for you in the future. The information gathered from this study will help us to find the best method to identify these people in the future. Some people also find that having the chance to talk to someone about their experience is helpful. If you have any questions or concerns about your diagnosis, the researcher will be able to pass this information back to your GP for them to help you. Your GP will be informed throughout the process and if you do have a lung condition then they will be able to manage your health better in the future.

Potential disadvantages: Taking part in the study will mean giving up some of your time, which may cause you some inconvenience. It is expected that visit 1 will take approximately 90 minutes and will be booked at a time that suits you. As part of visit 1 you will be asked to inhale a medication called Salbutamol which is a drug that is regularly used by patients with lung disease. The potential side effects of taking this drug include shaking, headache, a fast heart rate and cramp. These side effects are uncommon and should not last very long. The breathing tests may also make you feel dizzy, or may cause you to cough or be short of breath as you will be asked to take several deep breaths in and out. However, you will be seated while you complete the tests and the side effects should pass quickly.

Some people also find that talking about their symptoms or being told they are likely to have a new health condition upsetting. If you do feel upset you are welcome to discuss your feelings with the study team member during the visit, or we will advise you to make an appointment with your GP. Receiving a new diagnosis of a long term medical condition may affect life or holiday insurance policies that you may hold or are likely to take out in the future. This is common practice among insurance providers, and we would recommend that you declare all known medical conditions as a matter of course. This may concern some people, however please bear in mind that receiving a diagnosis and therefore having the right treatment and management plan for your condition is very important for your long term health. Should you have any concerns relating to this matter, you can contact the study team or your GP and we would be happy to answer any questions you may have.

Taking part in this study is completely up to you and you can withdraw from it at any stage if you so wish. If you decide not to carry on with the study, at no point will this impact upon your normal care from your GP practice team. If you have further concerns or worries we would recommend that you speak to your GP or contact the clinical research team (details at the end of this leaflet).

What happens to the data collected?
As part of the study, we will be collecting information about you from your medical records. Only information that is relevant to the study will be collected. This will include health records from your GP practice and any relevant hospital contact that you have had.

All information collected from you and your electronic medical records during the course of the study will be reviewed and analysed by the research team. No names or other such details that can identify you will be on any written information, to ensure and maintain strict confidentiality. The data collected will be stored on a password protected computer in the University of Southampton. Only the research team will have access to this information, this includes individuals from the CLAHRC Wessex team who are coordinating this project, as well as individuals from the University of Southampton and the University of Birmingham who are working with us on this project.

Information which could identify you (like your consent form) will not be stored with your data. Data will be analysed and used for this study only unless you give additional consent for your information to be used for other teaching/scholarly purposes. The findings from this study will be used in research reports and publications and at no time will your name appear in any such publications and any quotes used will be given a false name to protect your identity.

If you decide to take part in the telephone interviews your audio recording will be transcribed into a written format by the student researcher. Both the audio recording and the written transcription will be stored on a password protected computer in the University of Southampton. All written information obtained during the interview will be given a unique study number assigned to you at your first visit.

All data collected as part of the study will be stored for at least 10 years in line University of Southampton Policy.

What happens if I don't want to take part or I change my mind?
You do not have to take part in this research study. You can decide not to continue at any time without giving a reason and it will not affect your usual care from your GP in any way. If you decide to withdraw please contact the clinical research team (details at the end of this leaflet).

Who can I contact if I have a complaint?
If you have any concerns about any aspect of this trial please contact Dr Tom Wilkinson, Principal Investigator, on 02381206686 in the first instance. If you still have concerns, please contact the Research Governance Office at the University of Southampton (Phone: 02380 595058 Email: rgoinfo@soton.ac.uk) or you can complain formally through the NHS complaints procedure, Patient Advice and Liaison Service (PALS) (Phone: 02381206325 Email: patientsupportservices@uhs.nhs.uk). It is highly unlikely that you will be harmed during this study. If you are however, you may have grounds for legal action against the University of Southampton and the NHS.

What will happen to the results of the research study?
Once we have analysed the results we will publish them in a medical journal. We will also share them with other parts of the NHS such as other hospitals, and the results will also be shared with patients. The results may enhance treatment for future patients with lung diseases. A summary of the findings will be sent to you if you would like to see the results and the full report can be made available on request.

Who is organising and funding the research?
This study has been organised by the University of Southampton, the Collaboration for Leadership in Applied Health Research and Care (CLAHRC Wessex, Theme 1 Respiratory) team and in collaboration with West Hampshire CCG. The study is funded by a grant from the National Institute for Health Research (NIHR).

Who has reviewed the study?
The study has been reviewed by an independent academic expert in qualitative methodology, Patient and Public Involvement (PPI) representatives and the Collaboration and Leadership in Health Research and Care (CLAHRC) review board. The study has been given a favourable ethical opinion for conduct by NHS Research Ethics Committee South Central Hampshire B; reference 16/SC/0629.

Where can I get more information?
If you would like more information or to take part in the study, please do not hesitate to contact the clinical research team on:

Phone: **07867142279** Email: UHS.AssistStudy@nhs.net

The CLAHRC Research Team:

Dr Tom Wilkinson	*Associate Professor and Honorary Consultant in Respiratory Medicine*
Dr Mike Thomas	*GP and Professor of Primary Care and Population Sciences*
Helen Kruk	*Nurse Team Lead and Project Manager / Research Fellow*
Emma Ray	*Respiratory Nurse Specialist*
Kate Gillett	*Respiratory Nurse Specialist*
Mal North	*Respiratory Nurse Specialist*

THANK YOU FOR TAKING THE TIME TO READ THIS INFORMATION

BLANK PAGE

The ASSIST Study

Participant Initial JS Participant ID number CF/ 400

Consent Form

Study Title: A clinical interventional study into airways disease case finding and 'at risk' case management

Please Initial boxes

1. I confirm that I have had the study explained to me by the researcher and have read and understood the information sheet dated………………… version……… and have been given the opportunity to ask questions that have been answered satisfactorily. JS

2. I understand that all my details will be kept confidential and my name will not appear on any reports or documents. JS

3. I understand that my participation is voluntary and that I am free to withdraw at any stage without giving reasons, without my legal rights being affected. JS

4. I understand that information about me, where relevant to the study, will be stored by the CLAHRC Wessex clinical research team at the University of Southampton and Southampton University Hospital. JS

5. I consent to the collection and use of information bout me in accordance with the participant information sheet. I understand that relevant sections of my medical notes and data collected during the study may be looked at by responsible individuals from the CLAHRC Wessex team plus related NHS bodies and NHS regulatory authorities, as well as the Sponsor, the University of Southampton or Sponsor delegates from the University Hospital Southampton and the University of Birmingham or by regulatory authorities where it is relevant to my taking part in research. I give permission for these individuals to have access to my records in both primary and secondary care. JS

6. I give my permission to authorise personnel from the clinical research team to inform my General Practitioner of my participation in, and any relevant test results from this study. JS

7. I give my permission to be contacted in the future for related research purposes including other studies in the CLAHRC Wessex Respiratory programme. JS

EXAMPLE

8. I am willing to participate in a telephone or face to face interview at a later date, as explained in the participant information sheet. JS

9. I agree to take part in this study. JS

10. I would like to receive a summary of the study results when the research is complete and give the research team permission to send this to me by JS

i. Email √

ii. Post √

Jane Smith ______ Jane Smith ______ 12/06/2017

Name of Participant Signature Date

Annie Nurse ______ Annie Nurse ______ 12/06/2017

Name of Person taking Consent Signature Date

NB: x1 signed copy for researcher; x1 signed copy to patient: 1 x signed copy for medical records

EXAMPLE

Supplementary Methods

Twelve intervention practices were individually matched to control practices within the Optimum Patient Care Research Database (OPCRD). OPCRD contains the computerised records for a sample of over 700 general practices in the UK comprising over 7.3 million patients.

Only practices with data available for the entire intervention and follow up period were eligible to be matched. We used a pragmatic, calliper-based matching method to compare several practice-level indicators which are described in **Supplementary Table 1.**

We used a two-phased matching approach, using a wider interval for our calliper to find a short-list of practices for which the six variables in Table 1 lay within the 'first phase' calliper intervals. For ten of the twelve intervention practices the first phase was insufficient to provide a single match to use as a control practice, requiring use of the second phase (narrower) calliper intervals to iteratively tighten the thresholds using three of the matching variables which were deemed to be key among the six: prevalent smoking status; diagnosis of asthma and diagnosis of COPD.

In the few cases (3 out of 12) where a unique candidate to use as a matched control practice could not be found, one was randomly selected via a simple random sample with equal probability of selection.

All matching variables were defined using a clinician-validated set of Read codes (defined in Josephs *et al*, Eur Respir J, 2017) and applied to patient primary care records within OPCRD. Read codes are commonly used to record clinical activity in this care setting (Benson, J Innov Hlth Inf, 2011).

Supplementary Table 1: Practice matching thresholds

Indicator	Threshold	
	**First phase**	**Second phase [c]**
COPD prevalence	+/- 0.5%	+/- 0.2%
'Ever' smoker prevalence [a]	+/- 5% [b]	+/- 1.5%
Asthma prevalence	+/- 5%	+/- 2%
Proportion aged 40 years or over	+/- 7%	Not used
Total practice population	+/- 25% [b]	Not used
IMD decile	+/- 2 deciles	Not used

[a] Defined as the total number of patients with either ex- or current smoker status
[b] First phase thresholds relaxed to +/- 40% total practice population and +/- 12% 'ever' smoker prevalence for one intervention practice owing to lack of suitable matching practices
[c] Second phase indicators listed in order of precedence
[d] At any time up to data extraction

Supplementary References

1. Benson T. The history of the Read codes: the inaugural James Read Memorial Lecture 2011. Journal of Innovation in Health Informatics. 2011;19(3):173-82.

2. Josephs L, Culliford D, Johnson M, Thomas M. Improved outcomes in ex-smokers with COPD: a UK primary care observational cohort study. Eur Respir J. 2017;49(5).
